# Harnessing the Benefits of Telehealth in Long COVID Service Provision

**DOI:** 10.3389/phrs.2024.1606948

**Published:** 2024-05-31

**Authors:** Naomi Whyler, Liz Atkins, Prue Hogg, Amanda Leong, Julie Metcalfe, Michelle Scoullar, Emma Tippett

**Affiliations:** Clinic Nineteen Long COVID Clinic, Melbourne, VIC, Australia

**Keywords:** long COVID, telehealth, equity, care model, post COVID-19 condition

Dear Editors,

We commend Luo et al. for their in-depth analysis of the current available services in Australia to support people with Long COVID [1]. This devastating illness affects over 10% of those after acute COVID-19 infection and is projected to affect over 200 million people in the next decade worldwide [2]. Many with Long COVID are severely impacted by physical symptoms to the extent that simple activities of daily living are extremely fatiguing, and the demands of travel to a healthcare appointment can trigger episodes of severe post-exertional malaise which has been found to affect over 80% of those with Long COVID [3]. Provision of healthcare should be available in a format that does not worsen symptoms nor impact upon them financially.

**FIGURE 1:**
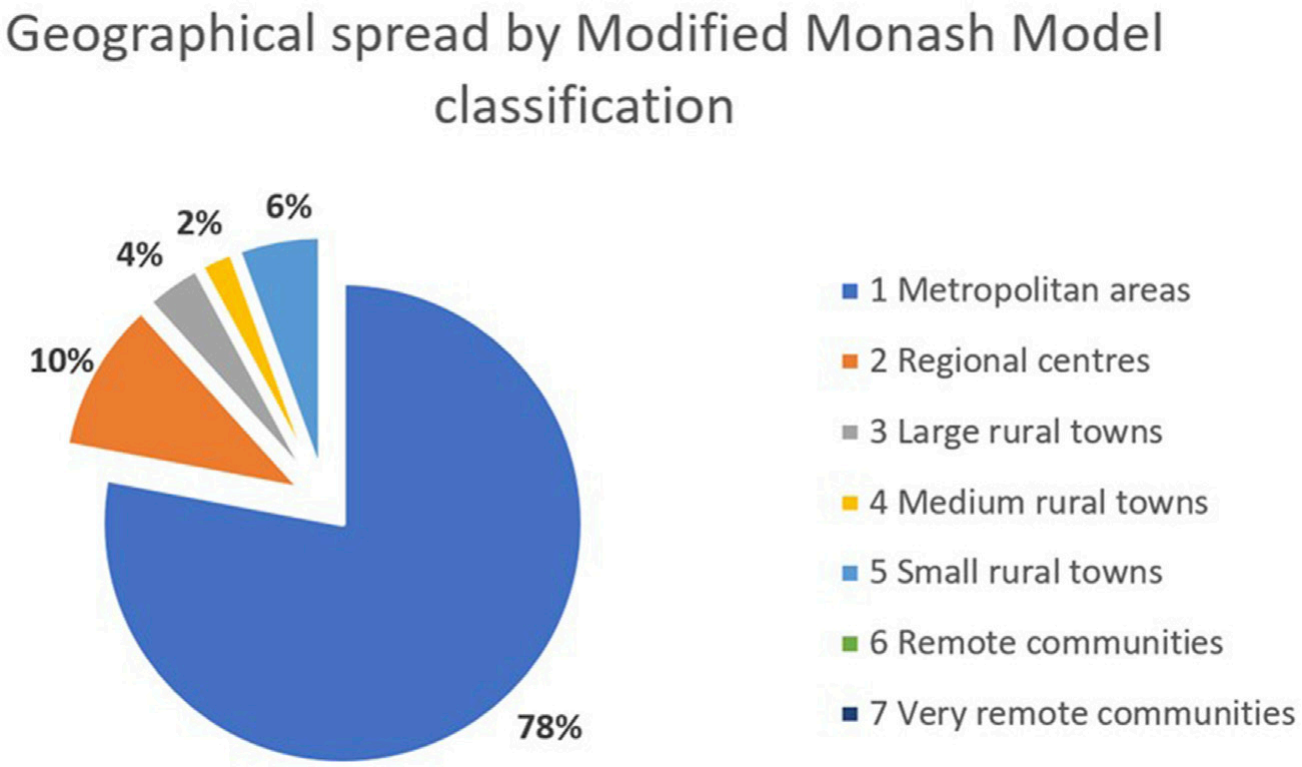
Geographical spread of cohort by Modified Monash Model [5] classification (unpublished data) (Footnote [1]) (Australia. 2023). *Modified Monash (MM) category 1: metropolitan areas in major cities accounting for 70% of Australia’s population; MM2: regional centres within 20 km road distance of town with population over 50,000; MM3: large rural town within 15 km of town with population 15,000 to 50,000; MM4: medium rural town within 10 km road distance of a town with population 5,000 to 15,000; MM5: small rural town, all other areas excluding MM6 and MM7 (remote and very remote communities).

As Luo et al. describe, options for Long COVID specialist care in Australia are limited, particularly for people in rural or remote locations [1]. We note, however, that their summary did not review the option of telehealth as a model of care for Long COVID. Telehealth encompasses provision of medical assessment, diagnosis, treatment, and education through the use of technology, including video and telephone-based consultation [4]. Our Australian-based clinic, which was not included in Luo et al.’s review, uses a telehealth model of care and to date has provided care to over 500 people with Long COVID (including children) from all states and territories, including the Northern Territory, which has no other dedicated Long COVID services [1]. Of our cohort, 22% live outside of major metropolitan centres as measured by the Modified Monash model [5] (Figure 1).

This model of care provides an option for patients with physical [6] or other disability and geographical limitations [7] to equitably access healthcare without physical detriment or disproportionate financial penalty due to travel costs. The need, strengths, safety and limitations of telehealth services to provide rapid and accessible care has been highlighted throughout the COVID-19 pandemic. Systemic changes within the Australian health system provided funding of a wide-scale shift in the modality of care delivery [4], and which have been trialled elsewhere including Canada [8].

The use of telehealth, where service is otherwise limited, provides a real option for many patients to receive care they would not otherwise be able to access [6], and the inability to undertake a physical examination can often be mitigated through close collaboration with the person’s primary care provider. This approach has been successfully demonstrated in several settings including with rehabilitation [9], an important facet of long COVID care. Furthermore, formal and informal consumer feedback from our clinic indicates that this model of care is desired by many people with Long COVID, in keeping with published literature [10]. Luo et al. highlight the importance of consumer engagement and empowerment, and including consumers in discussion about models of care is of paramount importance to be able to provide optimal quality care.

Provision of care for Long COVID must be equitable, should not exacerbate symptoms, and should be designed with consumer needs and opinions at its heart. The benefits of telehealth are numerous for those with Long COVID and should be embedded within systemic strategies to enhance care.
